# Glyphosate Residues in Groundwater, Drinking Water and Urine of Subsistence Farmers from Intensive Agriculture Localities: A Survey in Hopelchén, Campeche, Mexico

**DOI:** 10.3390/ijerph14060595

**Published:** 2017-06-03

**Authors:** Jaime Rendón-von Osten, Ricardo Dzul-Caamal

**Affiliations:** Instituto EPOMEX, Universidad Autónoma de Campeche, Campus VI, Av. Héroe de Nacozari 480, 24070 Campeche, México; ricardodzul210@hotmail.com

**Keywords:** glyphosate, groundwater pollution, bottled drinking water, human urine, subsistence farmers

## Abstract

The use of pesticides in Mexican agriculture creates an interest in learning about the presence of these substances in different environmental matrices. Glyphosate (GLY) is an herbicide widely used in the state of Campeche, located in the Mayan zone in the western Yucatan peninsula. Despite the fact that GLY is considered a non-toxic pesticide to humans, its presence in water bodies through spillage, runoff, and leaching are a risk to human health or biota that inhabit these ecosystems. In the present study, glyphosate residues were determined in groundwater, bottled drinking water, and the urine of subsistence farmers from various localities of the Hopelchén municipality in Campeche. Determination of GLY was carried out using Enzyme-Linked Immunosorbent Assay (ELISA). The highest concentrations of GLY were observed in the groundwater (1.42 μg/L) of Ich-Ek and urine (0.47 μg/L) samples of subsistence farmers from the Francisco J. Mújica communities. The glyphosate concentrations in groundwater and bottled drinking water indicate an exposure and excessive use of glyphosate in these agricultural communities. This is one of the first studies that reports glyphosate concentration levels in human urine and bottled drinking water in México and in the groundwater in the Yucatan Peninsula as part of a prospective pilot study, to which a follow-up will be performed to monitor this trend over time.

## 1. Introduction

The main crop in the state of Campeche is maize. About 144,000 hectares were cultivated in 2006, and just over 181,000 hectares in 2013 [1]. In addition to maize, rice, sugar cane, sorghum, jalapeño peppers, habanero peppers, and beans are principal crops in the region.

Aside from maize production, Campeche is the primary soybean producing state in Mexico [2]. According to current federal figures, the harvested areas and annual production have been growing from 2013–2014. Soybean cultivation doubled (29,200 hectares), with the Hopelchén municipality serving as the main soybean producer in the state [3]. Ninety percent of the soybean crop produced in Hopelchén comes from commercial and industrial producers, while the remaining percentage comes from subsistence farmers (Agricultural producers under the slash and burn model, mainly for self-consumption) [4]. In Mexico, the production by commercial operators has led to various criticisms due to the severe deforestation process, excessive use of agro-chemicals, and the use of genetically modified (GM) seeds; produced and mainly distributed by Monsanto, which are resistant to glyphosate herbicide application [5,6].

The main cause of deforestation in the study sites around Hopelchén comes from the expansion of industrial agriculture, accounting for 68% of the forest area lost (75,400 ha). This type of production is present throughout the municipalities, however, it is primarily found in sites with a slight slope, causing a high percentage of deforestation per municipality throughout the state of Campeche from 2000–2013 [7]. Annual mean rainfall is approximately 1100 mm. During some crop seasons the fields flood, therefore farmers often drill infiltration wells, to prevent flooding. Infiltration wells are constructed to induce the indirect filtration of water accumulated on the surface of the soil substrate [8]. The Yucatan peninsula is primarily covered with karstic soils, which are extremely permeable; as a result, the infiltration wells directly contaminate the groundwater with pesticides. At the study sites near Hopelchén, there are many illegal absorption wells that are not constructed to code, legally permitted, nor sealed. Water infiltration wells are a serious problem in the region because they contribute to desertification. In 2015, observations reported that the lagoons of Cancabchén and that the Ik Lagoon nearly dried up due to the loss of runoff waters [9].

The intensive use of pesticides in Hopelchén for sorghum, tomato, maize, and other crops causes groundwater contamination. Many pesticides have a half-life of several months, which allows them to persist for a long period in the environment. A study conducted in 2010 showed the presence of 2,4-dichlorophenoxyacetic acid (2,4-D) residues in the groundwater at some localities in the state of Campeche, including Hopelchén with 13.8 μg/L [10]. Pollution in the groundwater from pesticides is critical in karstic areas because substances such as glyphosate with a very low octanol/water coefficient (K_ow_ = 0.00033), are highly soluble in water, reaching a persistence of up to 170 days at 25 °C under low light conditions, and extending to 315 days at 31 °C in darkness [11]; these temperature and light conditions are very common in the Yucatan groundwater system.

In Mexico, there is only one study about the presence of glyphosate in both surface water and groundwater from natural protected areas and agricultural areas in the state of Chiapas [12]. Authors found higher concentrations of glyphosate during the dry season, probably due to reduced dilution from precipitation. The concentrations of glyphosate in groundwater ranged from <0.13 to 18.43 μg/L and from <0.13 to 36.71 μg/L in superficial water (river). Similarly, in a study carried out over a period of 3 years in an area near a Maya mountain protected area in Belize, glyphosate residues were found in the phytotelmic water at seven sites where the range of glyphosate concentrations was from 0.22 to 1.7 μg/L [13]. The authors mention that at least part of the population acquires its drinking water from systems coming from the Maya Mountain Protected Areas, and therefore populations could be chronically exposed to glyphosate. This study suggests that pesticide drift is occurring in these areas, and its dispersion will reach many places depending on the water systems exposed.

A study conducted in 2004 regarding agricultural production methods and pesticide use among subsistence farmers in four rural communities of Campeche, including Ich-Ek, Crucero San Luis, and Suc-Tuc from the Hopelchén area [14], showed that acetylcholinesterase (AChE) activity in subsistence farmers was significantly lower (*p* < 0.05) than the mean activity determined from individuals in a reference group. In this study, carbamates, particularly carbofuran, seem to be more associated with the symptomatology of pesticide exposure than organophosphates.

Glyphosate (*N*-(phosphonomethyl) glycine) is the most widely used broad-spectrum herbicide worldwide and its use in agriculture has increased since its introduction in 1970. It is primarily administered for weed control outbreaks during land preparation or pre-harvest. Thus, genetically modified plants resistant to glyphosate were created for soybean and maize production, assuring that the crops were not affected in the presence of glyphosate [15,16].

Genetically modified soybean cultivation emerged in the Yucatán peninsula in 2001. When the first experimental field was planted in 2010, a total of 12,000 hectares were used as pilot fields for genetically modified soybeans. In 2012, soybean producers requested authorization from the Federal Government to extend the area of genetically modified soybean production to 60,000 hectares. The use of genetically modified products such as soybeans entails the indiscriminate use of glyphosate because the transgenic seed-glyphosate dependency cannot be separated.

Aside from leaching into water systems, pesticides cause mortality in bees [17]. In the USA, approximately 13 million dollars are lost annually from bee mortality [18]. Although this is not a focal concern of our study, it is important because many of these crops depend on pollination, and honey is an important economic good produced in Campeche. Bee mortality was observed near glyphosate treated crops between 2012 and 2013. Almost 2000 bee colonies died in the towns of Suc-Tuc and San Luis in the municipality of Hopelchén, Campeche [19]. Sublethal effects in bees constitute other adverse effects from glyphosate which influence the production of honey. These effects are important because laboratory tests with sublethal concentrations of glyphosate (1.25, 2.5, 5, and 10 ng active ingredient/bee) created a slight decrease of acetylcholinesterase activity of the bee [20].

Although the European Food Safety Authority (EFSA) [21] concluded that glyphosate is unlikely to pose a carcinogenic hazard to humans, the International Agency for Cancer Research (IARC) had classified glyphosate inside the 2A Category, which indicates “probably carcinogenic to humans” [22]. Some studies indicate a correlation between glyphosate and genotoxic, hormonal, enzymatic type [23,24,25,26], reproductive [24,27], and neurological [28] health risks to humans.

Humans can directly be exposed to glyphosate as operators, passers-by, or residents of glyphosate applied areas, through the food chain [23,29,30] or human water consumption [31]. Pharmacokinetic studies indicate that glyphosate absorbed orally is excreted unchanged, predominantly in urine [23], where it can be measured at μg/L levels [32,33]. Thus, urine provides a measure of recent exposure and is considered an ideal matrix in biomonitoring studies [29,33,34,35].

Soybean crop expansion in the State of Campeche [6] as well as in other Latin American and European countries, has negative effects on the environment, especially by polluting water, as well as in human and animal health by the generalized and frequent use of glyphosate [36,37]. The goal of this study was to determine the levels of glyphosate in groundwater samples, bottled drinking water, and in the urine of subsistence farmers in different communities around the municipality of Hopelchén, Campeche.

## 2. Materials and Methods

### 2.1. Study Sites and Sample Collection

**Figure 1:**
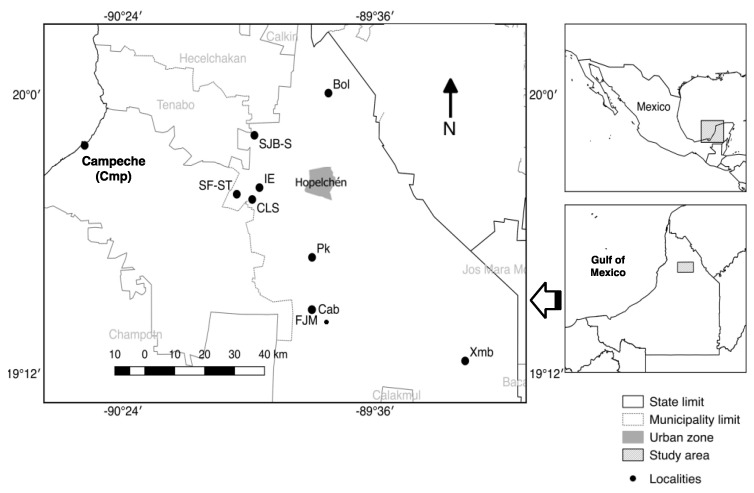
Sampling sites in the Municipality of Hopelchén, Campeche, Mexico. For details of the name of localities and the type and number of samples, refer to Table 1.

The Hopelchén municipality is located in the eastern part of the state of Campeche, close to the border with the state of Yucatan. Its geographical coordinates are 19°44′39″ N and 89°50′40″ W, with an area of 7460.27 km^2^. Hopelchén covers 13.1% of Campeche and is the most productive apicultural and agricultural municipality in the state (Figure 1). Groundwater, bottled drinking water, and urine from subsistence farmers were sampled from communities in the Chenes region, and the city of Campeche, an urban area used as a reference. This reference group consisted of male fishermen, not farmers (Figure 1). For a detailed description of the land use change in the area, we refer to the study by Ellis et al. [7].

From the five sites, 15 urine samples were collected from subsistence farmers at Ich-Ek (IE), Francisco J Mújica (F.J.M.), Suc-Tuc (SF-ST), San Juan Bautista Sahcabchén (SJB-S), and Crucero San Luis (CSL), and eight urine samples from fishermen at the City of Campeche, considered as a reference site (Ref) (Table 1).

### 2.2. Water Sample Collection

Two types of groundwater samples were collected; one was from wells that are used as water sources in the surrounding towns and the other was from infiltration wells drilled to drain excess water from fields during the rainy season.

Two of the water samples collected from the wells used as water sources were from Ich Ek and Suc-Tuc, one was from Sahcabchén, one was from Cancabchén, and six were from Campeche, the reference site. These samples were taken directly from the pump outlet. The samples from Ich-Ek, Bolonchén, Pakchén, Xmaben, Sahcabchén, and Cancabchén were collected from open wells. These samples were extracted with a reusable polyethylene Bailer sampler (1.6″ × 36″).

To produce bottled drinking water in the region, groundwater is extracted and treated by reverse osmosis. This drinking water is sold in large refillable five-gallon water containers.

Groundwater and bottled drinking water samples were collected, transported, and stored in dark conditions at 4 °C in 200 mL amber colored glass bottles, completely cleaned with diluted HCl acid and washed with ultra-pure water [12]. In the laboratory, the samples were separated (1 mL) into sterile Eppendorf tubes and cooled in dark conditions at 4 °C, until their analysis. Because only two localities have water treatment plants in the area, four samples were collected from Ich-Ek, two from Bolonchén, and seven from the city of Hopelchén, and two samples from the city of Campeche were included in the analysis as a reference site.

### 2.3. Urine Sample Participants

Morning urine samples were collected to perform a cross section prospective study from subsistence farmers in five communities from the Hopelchén area. Urine samples were provided in 60 mL polystyrene containers with polyethylene screw caps. The participants labeled the containers according to the collection date and the containers were stored in coolers for further analysis [38]. All of the samples were provided by subsistence farmers who were adult men between 30 to 50 years of age, and had signed a written informed consent. The Research Committee at the Universidad Autónoma of Campeche approved the samples under project registration no. 095/UAC/2015, and the study was performed in accordance with the principles of the Declaration of Helsinki. The only inclusion criterion for the random sampling was that participants were subsistence farmers. Persons or groundwater samples not fulfilling the inclusion criterion had to be excluded with the exception of people from the reference group (fishermen). Urine samples were diluted with ultra-pure water (Barnstead Easypure, Barnstead/Thermolyne, Dubuque, IA, USA) at a ratio of 1:20. Fifty μL of urine and water samples were centrifuged at 10,000× *g* for 10 min at 20 °C and the supernatants were tested for glyphosate using Enzyme-Linked Immunosorbent Assay (ELISA) kits. Because the first urine samples were negative, researchers decided not to dilute the samples.

### 2.4. Glyphosate Analysis by ELISA

For the immunoanalytical detection of glyphosate, the ELISA method (PN 500086) by Abraxis LLC (Warminster, PA, USA) was employed by using a microplate reader (Multiskan Spectrum, Thermo Scientific, Waltham, MA, USA). Measurements were carried out according to the manufacturer’s instructions [39]. This method showed a detection limit (DL) of glyphosate in water of 0.05 μg/L, a quantification limit of (QL) of 0.13 μg/L, a maximum detectable concentration of 4 μg/L, and a mean recovery rate of 102 %. For quantification, a four-point calibration curve was constructed with two replicates with a blank measured as zero for comparison (0.00, 0.075, 0.2, 0.75, and 4 μg/L). An analytical quality control solution was used with 0.75 μg/L of glyphosate provided with the Abraxis Glyphosate Plate Assay kit. According to the analytical results of Byer at al. [40], they found an R2 value of 0.88, which indicates a close relationship between Liquid Chromatography/Tandem Mass Spectrometry (LC/MS/MS) and ELISA; however, the authors mention that ELISA tends to overestimate the concentration of glyphosate present. This overestimation could be explained by the presence of matrix interferences such as cross-reactive related analogues, organic matter, salts, or phosphates, or both. Conversely, Clegg et al. [41] and Rubio el al. [42] reported that the Abraxis glyphosate immunoassay has shown no reactivity to other substances such as some ions and humic acids. Nonetheless, a study conducted by Krüger et al. [43] validated the immunoassay with Gas Chromatography-Mass Spectroscopy (GC-MS) obtaining a correlation coefficient of 0.96 between the two tests. Mörtl et al. [44] quantified the levels of glyphosate in surface and groundwater in Hungary using the Abraxis kit. Their results showed high concentrations of glyphosate (almost 1 μg/L). High concentrations of this herbicide were attributed to agricultural activities, natural precipitation, and to a greater extent, catchment area characteristics, resulting in a variation of glyphosate leaching or run-off to surface waters. The literature also concludes that this method presents great advantages such as simplicity and specificity (cross-reactivity is below 0.1% for related compounds). Matrix effects were also observed only in tap water, possibly due to chlorination or heavy metal ions, or both, e.g., copper and zinc. For a better comparison of the results, the analytical detection or quantification limits, or both, measured in the concentrations were expressed as 1 ppb = 1 μg/L.

### 2.5. Statistical Analysis 

To identify statistical differences, all analyses were performed by one-way ANOVA where the *p* value was set at *p* ≤ 0.05. Statistical analysis was performed using the program Graph Pad Prism, version 6.0 (www.graphpad.com) (Graph Pad Software Inc., San Diego, CA, USA) and Microsoft Excel 2010 (Microsoft, Albuquerque, NM, USA).

## 3. Results

### 3.1. Glyphosate in Groundwater

**Figure 2:**
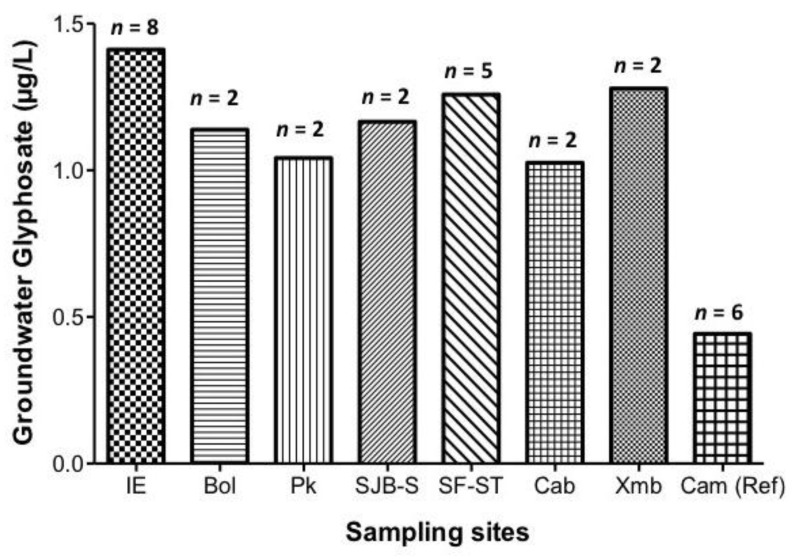
Glyphosate concentrations (μg/L) in the groundwater samples of the locations being monitored. Data are expressed as means. For details of the name of localities and the type and number of samples, refer to Table 1.

Glyphosate detection in groundwater samples presented variations, which depended on the sampling locations. Glyphosate was detected in all of the agricultural communities studied (Figure 2, and Supplementary Table S1). The groundwater samples from Ich-Ek presented the highest concentrations of 1.41 μg/L, followed by Xmaben (1.27 μg/L) and San Francisco Suc-Tuc (1.25 μg/L). The reference community in the city of Campeche showed the lowest levels of glyphosate in the groundwater samples (0.44 μg/L) (Figure 2).

### 3.2. Glyphosate in Bottled Drinking Water

**Figure 3:**
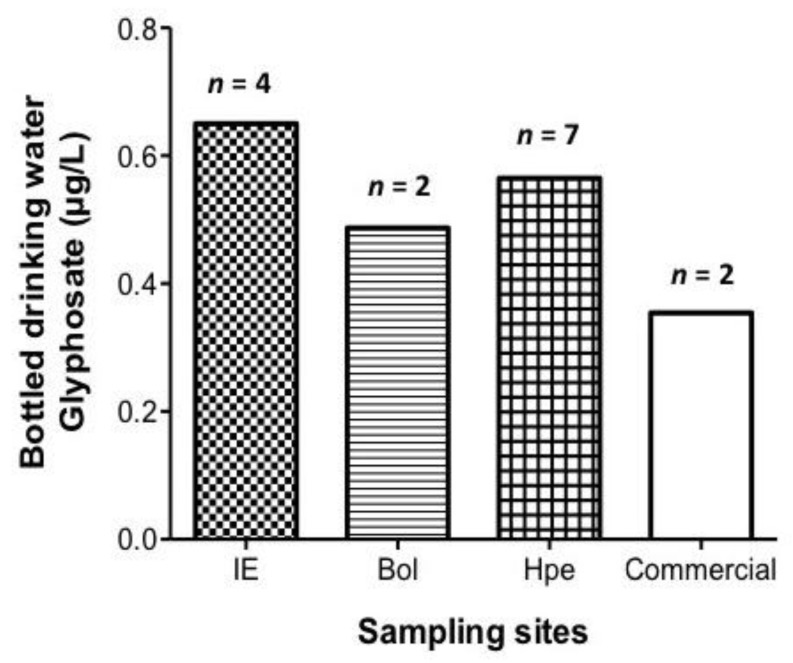
Glyphosate concentrations (μg/L) in bottled drinking water samples of the locations being monitored. Data are expressed as means. The samples named “commercial” comes from the city of Mérida.

Glyphosate was present in the bottled drinking water samples from the three communities monitored: Ich-Ek, Bolonchén, and Hopelchén; as well as in the commercially bottled drinking water from Mérida. The Ich-Ek community presented the highest mean levels of glyphosate (0.65 μg/L). Commercially bottled drinking water from Mérida presented the lowest mean glyphosate levels (0.35 μg/L) (Figure 3, and Supplementary Table S1).

### 3.3. Glyphosate in Human Urine

**Figure 4:**
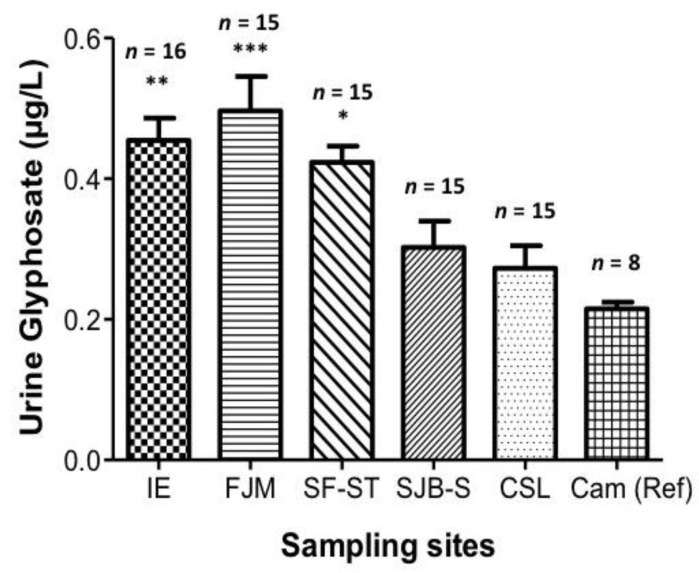
Glyphosate concentrations (μg/L) in urine samples of the locations being monitored. Data are expressed as means ± Standard Deviation (SD). Statistical differences are represented as * *p* ≤ 0.05, ** *p* ≤ 0.01, and *** *p* ≤ 0.001.

Eighty-one urine samples from subsistence farmers were analyzed. As demonstrated in Figure 4, the community in Francisco J. Mújica (F.J.M.) presented the highest mean glyphosate concentration value in urine at 0.47 μg/L (Figure 4). In contrast, the urine samples analyzed from the urban reference group (fishermen in Campeche) presented the lowest mean concentration (0.22 μg/L). Also, significant differences were observed (*p* < 0.05) among the urine samples from farmers of Ich-Ek (IE) and Francisco J. Mújica (F.J.M.), compared to the urine samples from residents of Crucero San Luis (CSL) and the fishermen from the city of Campeche (Reference) (Figure 4, and Supplementary Table S1).

## 4. Discussion

### 4.1. Residual Levels of Glyphosate in Groundwater Samples with Intense Agricultural Activity

The widespread use and application of glyphosate in agricultural and urban areas results in residues in crops, water ecosystems, and human urine samples, putting human health at risk [16,43,45,46].

Few studies have been conducted to determine glyphosate residues in ground water. The results obtained in previous studies have shown significant concentrations of glyphosate [47].

Our results indicate the presence of glyphosate in 90% of all groundwater samples evaluated, where Ich-Ek, Xmaben, and San Francisco Suc-Tuc had the highest glyphosate concentrations. The concentrations obtained in these communities (between 0.47 μg/L and 1 μg/L) were above the threshold of 0.1 μg/L reported in groundwater permitted by the European Glyphosate Task Force (GTF) [48]. Kaiser et al. [13] documented glyphosate concentrations of 0.221 μg/L to 1.708 μg/L in groundwater samples surrounding a protected area of the Maya Mountains in Belize. Mörtl et al. [44] reported concentrations ranging from 0.54 to 0.76 μg/L in groundwater samples in Hungary, and Sanchís et al. [46] reported concentrations of 2.5 μg/L in groundwater from Catalonia (Spain).

Although our results showed high levels of glyphosate in water samples, these results were lower in comparison to a study conducted in Chiapas, Mexico by Ruiz-Toledo et al. [12], who found high concentrations of glyphosate (36.7 μg/L) in the groundwater near agricultural crops. Reports in other countries by Davis et al. [49] found 54 μg/L of glyphosate in Australia, Van Stempvoort et al. [50] found 40.8 μg/L in southern Ontario (Canada), and Peruzzo et al. [51] found 700 μg/L in Argentina. The Canadian Water Quality Guideline for glyphosate for the protection of freshwater aquatic life is 65 μg/L [40]; though this guideline does not apply to Mexico, it suggests that the glyphosate residues in the water sampled in this study from the Yucatán Peninsula may not pose a threat to aquatic organisms.

The presence of glyphosate found in the groundwater samples in this study can be associated with sampling sites in proximity to glyphosate-resistant soybean fields, because it is documented that the Hopelchén municipality is one of the main soybean producers in the State of Campeche [3,6]. High glyphosate concentrations in groundwater near glyphosate-resistant soybean fields have also been documented in other studies [12,51,52].

The presence and frequency of high glyphosate concentrations in groundwater samples contrast in the literature, indicating the absorption and relatively fast degradation of glyphosate in soil [53,54,55]. The karstic soil characteristics in the Yucatán Peninsula indicate that this herbicide can be persistent for long periods of time in groundwater ecosystems [11]. Borggaard and Gimsing [56] found that in structured soils with macropores, such as karstic soils, preferential flow may carry glyphosate to drainage systems, and heavy rainfall shortly after glyphosate application seems to suggest that this herbicide has a high risk of leaching in high quantities to the groundwater in Yucatán; in addition to the accumulation of this herbicide through the infiltration wells in crop fields, which are receiving pesticide application.

Glyphosate residues in groundwater also depend on how they are incorporated. For example, in the karstic zone, it has been demonstrated that during storm events, pollutants adhered to the soil surface, and were concentrated and discharged during the rapid conduit flow portion of the hydrograph [57]. The Yucatán Peninsula karstic aquifer is vulnerable to pollution because this area has large amounts of groundwater sources, maintaining highly dependent ecosystems [58].

The evidence has shown that most glyphosate monitoring studies in aquatic ecosystems have focused on agricultural areas [46,59,60,61], suggesting the generalized and global use of glyphosate as an herbicide in agriculture [62,63]. Another source of glyphosate pollution could be the concentration of this compound in water sources from rainfall [64].

### 4.2. Residual Levels of Glyphosate in Bottled Drinking Water Samples

Almost all bottled drinking water samples in this study exceeded the acceptable limits of glyphosate for human consumption in the European Union (0.1 μg/L) [65]. It is important to note that in Mexico, there are no specific acceptable limits for glyphosate in drinking water; however, the Mexican Standard NOM-201-SSA1-2015 [66] states that non-halogenated organic compounds, including glyphosate, should not exceed 10 μg/L. In this case, none of the samples exceeded the maximum permitted level of the NOM-201-SSA1-2015.

### 4.3. Residual Levels of Glyphosate in Human Urine Samples

Urine is an important elimination route for glyphosate. Thus, it is not surprising that certain amounts can be detected in human urine samples, especially if glyphosate is absorbed through direct exposure from agricultural practices, in the diet, or in drinking water [33].

In the present study, the mean glyphosate concentrations in urine samples for people living and working inside the agricultural areas of Francisco J. Mújica (F.J.M.) were higher than those that lived in the city of Campeche (0.47 μg/L and 0.22 μg/L, respectively). These results were higher than those reported in first world countries such as Switzerland (0.16 μg/L). The results obtained in our study were lower than other studies from both the United States and Europe (1.33 μg/L–1.82 μg/L), respectively [29,34,35].

The differences found in our results from those reported in other studies, likely reflect the differences between glyphosate use in agricultural fields and crop plantations with genetically modified seeds in other countries, such as maize and cotton, as well as the frequent use of this herbicide. The differences also likely reflect the frequency with which this herbicide is applied. Niemann et al. [35] and Kruger et al. [34], suggested that a trend towards the increase of glyphosate concentrations in urine samples in the European population probably reflects more frequent use of glyphosate in agricultural practices. Other studies have documented that glyphosate causes an ecological risk to aquatic ecosystems [54,67,68], and can be bio-accumulated in aquatic organisms, thus placing human health at risk for those who consume these products [66,69,70].

## 5. Conclusions

This is the first report regarding the presence of glyphosate in water and urine samples in subsistence farmers inhabiting different agricultural communities in the State of Campeche, Mexico. The concentrations and glyphosate ratios in the matrices evaluated indicate a high exposure to this pesticide. It is very important to conduct a more detailed follow-up study to understand the presence of glyphosate in bottled drinking water, correlated with the glyphosate concentrations in subsistence farmers that used this herbicide, since there is no evidence using this matrix. This is of concern because it could explain another possible route of exposure to this pesticide. The presence of glyphosate in urine is very important even at low concentrations because glyphosate puts human health at risk. Therefore, the potential ecological impact of this pesticide must be considered on a more global, rather than regional, scale.

The results indicate the need to establish a monitoring program for pesticide residues in the Yucatán Peninsula. They also suggest a need to monitor non-target organisms, such as bees, to establish the effect of the predominant agricultural practices currently occurring in Hopelchén.

Also relevant to humans, it is essential to carry out a study that considers the health status of the human population in Hopelchén because glyphosate is among the dozens of widely used pesticides in the industrialized agricultural area. Due to the large cultivation areas and the quantities of pesticides applied, exposure to these chemicals by the population and field workers in the area should be evaluated to avoid diseases associated with these compounds.

## Figures and Tables

**Table 1:** Name of the localities where groundwater, bottled drinking water, and urine from subsistence farmers were sampled.

Localities	ID	Coordinates		Samples		
		Latitude N	Longitude W	Ground Water	Bottled Drinking Water	Urine
				*n* = 29	*n* = 15	*n* = 84
Ich-Ek	IE	19°43′59.8″	89°58.0′0.1″	8	4	16
Bolonchén	Bol	20°00′150″	89°44′51.0″	2	2	
Pakchén	Pk	19°31′58.8″	89°47′60.0″	2		
SJB Sahcabchén	SJB-S	19°52′58.8″	89°58′58.8″	2		15
SF Suc-Tuc	SF-ST	19°42′50.4″	90°02′20.4″	5		15
Cancabchén	Cab	19°22′59.8″	89°47′60.0″	2		
Xmaben	Xmb	19°14′09.6″	89°18′50.4″	2		
Francisco J. Mújica	F.J.M.	19°18′43.5″	89°44′22.5″			15
Crucero San Luis	CSL	19°41′56.4″	89°59′24.0″			15
Hopelchén	Hp	19°44′41.0″	89°50′42.0″		7 *	
Campeche (** Ref)	Cmp	19°50′55.0″	90°31′31.0″	6	2	8

*n*: total samples of each type. * Two samples of these were bought in Hopelchén but come from Merida City; ** Ref: Reference; ID: identity code; N: north; W: west.

## Supplementary Materials

The following are available online at www.mdpi.com/1660-4601/14/6/595/s1, Table S1: Glyphosate concentrations.
