# Supplementary material for: Glyphosate Residues in Groundwater, Drinking Water and Urine of Subsistence Farmers from Intensive Agriculture Localities: A Survey in Hopelchén, Campeche, Mexico
Source: Int J Environ Res Public Health. 2017 Jun 3;14(6):595. doi: 10.3390/ijerph14060595 (PMC5486281; doi:10.3390/ijerph14060595)
Supplement: Supplementary file 1 [file ijerph-14-00595-s001.pdf]

Table S1. Concentrations of glyphosate (ppb) in groundwater, drinking water and urine from Hopelchen, Campeche

**Localities**	**Groundwater** **Samples**	**ppb de glifosto**
Ich-Ek (IE)	Groundwater	1.4926
Ich-Ek (IE)	Groundwater	1.3399
Ich-Ek (IE)	Groundwater	1.5758
Ich-Ek (IE)	Groundwater	1.5438
Ich-Ek (IE)	Groundwater	0.9828
Ich-Ek (IE)	Groundwater	1.1788
Ich-Ek (IE)	Groundwater	1.4711
Ich-Ek (IE)	Groundwater	1.7056
Bolonchén (Bol)	Groundwater	1.1115
Bolonchén (Bol)	Groundwater	1.1652
Pakchén (Pk)	Groundwater	1.0269
Pakchén (Pk)	Groundwater	1.0572
SJB Sacabchén (SJB-S)	Groundwater	1.0984
SJB Sacabchén (SJB-S)	Groundwater	1.2329
Cancabchén (Cab)	Groundwater	1.1039
Cancabchén (Cab)	Groundwater	0.9481
Xmaben (Xmb)	Groundwater	1.4132
Xmaben (Xmb)	Groundwater	1.1443
SF Suc-Tuc (SF-ST)	Groundwater	1.1914
SF Suc-Tuc (SF-ST)	Groundwater	1.3259
SF Suc-Tuc (SF-ST)	Groundwater	N.D.
SF Suc-Tuc (SF-ST)	Groundwater	N.D.
SF Suc-Tuc (SF-ST)	Groundwater	N.D.
Campeche (Cmp (Ref))	Groundwater	0.6323
Campeche (Cmp (Ref))	Groundwater	0.1674
Campeche (Cmp (Ref))	Groundwater	0.7072
Campeche (Cmp (Ref))	Groundwater	0.2642
Campeche (Cmp (Ref))	Groundwater	0.2642
Campeche (Cmp (Ref))	Groundwater	0.2642
Positive control glyphosate		0.7288
Positive control glyphosate		0.7548
Positive control glyphosate		0.7464

Bottled drinking water

**Localities**	**Samples**	**ppb de glifosto**
Bolonchén (Bol)	Bottled drinking wa	0.4736
Bolonchén (Bol)	Bottled drinking wa	0.5005
Hopelchén (Hp)	Bottled drinking wa	0.5161
Hopelchén (Hp)	Bottled drinking wa	0.5608
Hopelchén (Hp)	Bottled drinking wa	0.6943
Hopelchén (Hp)	Bottled drinking wa	0.4750
Hopelchén (Hp)	Bottled drinking wa	0.5781
Ich-Ek (IE)	Bottled drinking wa	0.5173
Ich-Ek (IE)	Bottled drinking wa	0.5343
Ich-Ek (IE)	Bottled drinking wa	0.7883
Ich-Ek (IE)	Bottled drinking wa	0.7608
Commercial	Bottled drinking wa	0.3864
Commercial	Bottled drinking wa	0.3221
Commercial	Bottled drinking wa	0.0355
Commercial	Bottled drinking wa	N.D.
Positive control glyphosate		0.7612
Positive control glyphosate		0.7396

Urine

**Localities**	**Samples**	**ppb de glifosto**
Ich-Ek (IE)	Urine	0.3716
Ich-Ek (IE)	Urine	0.3122
Ich-Ek (IE)	Urine	0.3266
Ich-Ek (IE)	Urine	0.4220
Ich-Ek (IE)	Urine	0.4344
Ich-Ek (IE)	Urine	0.8429
Ich-Ek (IE)	Urine	0.5105
Ich-Ek (IE)	Urine	0.3422
Ich-Ek (IE)	Urine	0.3655
Ich-Ek (IE)	Urine	0.4020
Ich-Ek (IE)	Urine	0.4544
Ich-Ek (IE)	Urine	N.D.

Ich-Ek (IE)	Urine	N.D.
Ich-Ek (IE)	Urine	N.D.
Ich-Ek (IE)	Urine	N.D.
Ich-Ek (IE)	Urine	N.D.
Francisco J. Mújica (FJM)	Urine	0.2543
Francisco J. Mújica (FJM)	Urine	0.3836
Francisco J. Mújica (FJM)	Urine	0.3084
Francisco J. Mújica (FJM)	Urine	0.3219
Francisco J. Mújica (FJM)	Urine	0.3021
Francisco J. Mújica (FJM)	Urine	0.5566
Francisco J. Mújica (FJM)	Urine	0.5948
Francisco J. Mújica (FJM)	Urine	0.5037
Francisco J. Mújica (FJM)	Urine	0.8754
Francisco J. Mújica (FJM)	Urine	0.4940
Francisco J. Mújica (FJM)	Urine	0.3447
Francisco J. Mújica (FJM)	Urine	0.3786
Francisco J. Mújica (FJM)	Urine	0.7959
Francisco J. Mújica (FJM)	Urine	N.D.
Francisco J. Mújica (FJM)	Urine	N.D.
SF Suc-Tuc (SF-ST)	Urine	0.3298
SF Suc-Tuc (SF-ST)	Urine	0.4793
SF Suc-Tuc (SF-ST)	Urine	0.4141
SF Suc-Tuc (SF-ST)	Urine	0.3133
SF Suc-Tuc (SF-ST)	Urine	0.2444
SF Suc-Tuc (SF-ST)	Urine	N.D.
SF Suc-Tuc (SF-ST)	Urine	N.D.
SF Suc-Tuc (SF-ST)	Urine	N.D.
SF Suc-Tuc (SF-ST)	Urine	N.D.
SF Suc-Tuc (SF-ST)	Urine	N.D.
SF Suc-Tuc (SF-ST)	Urine	N.D.
SF Suc-Tuc (SF-ST)	Urine	N.D.
SF Suc-Tuc (SF-ST)	Urine	N.D.
SF Suc-Tuc (SF-ST)	Urine	N.D.
SF Suc-Tuc (SF-ST)	Urine	N.D.
SJB Sacabchén (SJB-S)	Urine	0.6884
SJB Sacabchén (SJB-S)	Urine	0.2427
SJB Sacabchén (SJB-S)	Urine	0.3076
SJB Sacabchén (SJB-S)	Urine	0.1046
SJB Sacabchén (SJB-S)	Urine	0.1312
SJB Sacabchén (SJB-S)	Urine	0.4972
SJB Sacabchén (SJB-S)	Urine	0.1023
SJB Sacabchén (SJB-S)	Urine	0.2652

SJB Sacabchén (SJB-S)	Urine	0.4131
SJB Sacabchén (SJB-S)	Urine	0.3078
SJB Sacabchén (SJB-S)	Urine	0.0641
SJB Sacabchén (SJB-S)	Urine	0.2875
SJB Sacabchén (SJB-S)	Urine	0.5968
SJB Sacabchén (SJB-S)	Urine	N.D.
SJB Sacabchén (SJB-S)	Urine	N.D.
Crucero San Luis (CSL)	Urine	0.2585
Crucero San Luis (CSL)	Urine	0.1464
Crucero San Luis (CSL)	Urine	0.3004
Crucero San Luis (CSL)	Urine	0.2193
Crucero San Luis (CSL)	Urine	0.2753
Crucero San Luis (CSL)	Urine	0.4707
Crucero San Luis (CSL)	Urine	0.1754
Crucero San Luis (CSL)	Urine	0.4105
Crucero San Luis (CSL)	Urine	0.1529
Crucero San Luis (CSL)	Urine	0.4905
Crucero San Luis (CSL)	Urine	N.D.
Crucero San Luis (CSL)	Urine	N.D.
Crucero San Luis (CSL)	Urine	N.D.
Crucero San Luis (CSL)	Urine	N.D.
Crucero San Luis (CSL)	Urine	N.D.
fishermen	Urine	0.2058
fishermen	Urine	0.1935
fishermen	Urine	0.2370
fishermen	Urine	0.2538
fishermen	Urine	0.1814
fishermen	Urine	0.2015
fishermen	Urine	N.D.
fishermen	Urine	N.D.
Positive control glyphosate		0.7283
Positive control glyphosate		0.7597
